# Analysis of the Concept of Obstetric Violence: Scoping Review Protocol

**DOI:** 10.3390/jpm12071090

**Published:** 2022-06-30

**Authors:** Ana Cristina Ferrão, Margarida Sim-Sim, Vanda Sofia Almeida, Maria Otília Zangão

**Affiliations:** 1 Comprehensive Health Research Centre (CHRC), Instituto de Investigação e Formação Avançada, University of Évora, 7002-554 Évora, Portugal; 2 Maternal Health and Obstetrics, Centro Hospitalar Barreiro-Montijo, EPE, 2830-003 Barreiro, Portugal; vandalmeida1@gmail.com; 3 Comprehensive Health Research Centre (CHRC), Nursing Department, University of Évora, 7002-554 Évora, Portugal; msimsim@ouevora.pt (M.S.-S.); otiliaz@uevora.pt (M.O.Z.)

**Keywords:** obstetric violence, woman, childbirth

## Abstract

(1) Background: Obstetric violence has been highlighted in the political and social agenda of several countries. Efforts have been made to create policies to humanize obstetric care, guarantee the rights of pregnant women and respond to this form of violence. The lack of consensus on the appropriate terminology to name and define the behaviours that constitute obstetric violence, hinders this process. (2) Objective: To analyse the concept of obstetric violence related to assistance to women during labor. (3) Methodology: Scoping review protocol, according to the Joanna Briggs Institute method. The search will be performed on EBSCOhost Research Platform, PubMed, Virtual Health Library and SciVerse Scopus databases. The Open Scientific Repository of Portugal will also be considered. All types of studies, published in the last 10 years, in English, Spanish and Portuguese languages, constitute inclusion criteria. Studies of women experiencing labor, in a hospital setting, that address the dimensions of the concept of obstetric violence will be reviewed. (4) Discussion: The results will serve as a basis for identifying the appropriate terminology of the concept of obstetric violence, in order to direct future research with interest in the problem.

## 1. Introduction

Traditionally, childbirth and its inherent care were ensured by women, popularly called midwives, who, through hands-on experience knowledge passed from generation to generation, were responsible for home care during birth. Childbirth was an intimate and familiar event, exclusive to the female universe [1]. Due to the evolution of scientific knowledge and technological advances, childbirth care has become a hospital-based practice with the goal of reducing maternal and infant mortality rates, and has become a public and medical event. However, international efforts created to increase the number of live births in health facilities and reduce childbirth-related maternal deaths, have led health professionals to neglect emotional and social aspects in their relationship with women, focusing mainly on the technological dimensions of the labor process [2].

The hospitalization of labor emerged associated with the biomedical model in obstetric care, connoting it as a pathological occurrence, marked by the need for medical intervention and management. Birth is no longer seen as a normal physiological process; women assume a passive role while health professionals control and intervene in childbirth [3]. Childbirth as a medical event came to represent the hegemony of health professionals, namely obstetricians and midwives, exercised over women as a way to ensure their professional and social status [2]. Therefore, the predominant model of care in birth care became centered in the hospital institution, instead of being centered in women’s needs, determining their permanent exclusion in the participation of decisions that involve them during labor [2,4].

The female body has become integrated as a medical right, being subjected to institutionalized procedures in the structures of health services, which are abusive and disrespectful, as well as to several unnecessary interventions, many of them with a violent configuration without proven scientific effectiveness, which can consequently bear greater risks, compromising maternal-fetal safety and well-being [4].

Labor is one of the moments in which women are most vulnerable, requiring attention, assistance, and care. However, this moment is often permeated by violence in hospitals, practiced precisely by those who should be their main caregivers, the health professionals [5]. The experience of childbirth determines the exposure of women, sometimes with loss or inability of their own autonomy in relation to their bodies, facing medical practices and established social norms [6].

The understanding of this type of violence is based on the established relational paradigms, characterized by unequal power relations between women and care providers, based on technical and scientific knowledge, as well as on the cultural and moral authority assigned to health professionals. When this asymmetry of power between them becomes a hierarchical relationship with the purpose of limiting or preventing women’s autonomy, violence is established.

Childbirth represents one of the most significant human experiences, with the potential to impact women in both positive and negative ways. Although the moment of birth is idealized as a positive experience, with a humanizing and respectful character, the fear of pain and the dehumanization of care by healthcare professionals are problems highlighted by women regarding the fear of childbirth [7].

The lack of information and consent to the procedures performed, as well as the limitation of the right to participate in the decision-making process regarding labor; insufficient or unassured pain relief; lack of trust and safety due to dehumanizing attitudes and submission to routine interventions by health professionals; and the experience of abuse, including threats of violence by professional midwives, are factors listed by women in the traumatic experience of childbirth [8].

Numerous scientific evidence suggests that labor and especially childbirth in health care facilities are associated with various levels of mistreatment, abuse, disrespect, neglect, and violence, affecting a significant number of women worldwide [9,10], with repercussions on their health and impact on the perceived quality of care received [11]. Contrary to what we might think, violence in childbirth does not occur exclusively in underdeveloped countries, being a reality that also affects developed countries or those considered to have a high per capita income, which reflects the global dimension of this phenomenon, as a serious public health problem [12].

The immediate consequences for women’s health, associated with mistreatment and abusive behavior in childbirth, are related to unnecessary procedures and interventions which do not respect the physiology of labor and can determine an increased risk of cesarean section and complications such as vaginal trauma, postpartum hemorrhage, and physical lesions due to abdominal pressure exerted by health professionals as a way to shorten the expulsion period [11]. Difficulties in the emotional and sexual relationship between the couple, as well as in the establishment of the emotional bond between mother and child, with impaired breastfeeding, occur in women with postpartum depression or post-traumatic stress disorder, resulting from childbirth experiences marked by disrespect and abuse, which is exercised either by obstetricians or professional midwives [11,13]. Moreover, in the long term, traumatic memories of labor may limit the desire for a new pregnancy or reduce satisfaction and trust in health care professionals, dissuading women from accessing health care systems in future pregnancies, with serious consequences for maternal-fetal well-being [11,14].

In the context of violence in health institutions, and specifically violence against women during labor assistance, the concept of obstetric violence undoubtedly emerges in the media debate that assists it. The terminology obstetric violence emerged in Latin America and Spain in the 2000s, through the activist movements for the humanization of childbirth, in an attempt to rescue women’s autonomy in the process of parturition [15]. Venezuela was the pioneer country in the construction of the term, as well as its legal definition, in 2007. This definition considers obstetric violence as any behavior, action or omission triggered by the team of health professionals, directly or indirectly, in public or private context, characterized by the domination of the woman’s body, as well as her reproductive processes, which is manifested by a dehumanized assistance, abuse of medicalization and pathogenesis of the reproductive physiological processes, resulting in the loss of the woman’s autonomy and her capacity of free decision, negatively impacting her quality of life and well-being [16].

Other Latin American countries, namely Argentina in 2009 and Mexico in 2014 have also recognized obstetric violence as a form of violence against women, punishable by law. In other American, Asian, African or European countries, the expression of obstetric violence has been taking shape, due to several reports from healthcare entities, which emphasizes the magnitude of this problem, however, there is no specific judicial law [17], that refers to Portugal.

In 2014, the World Health Organization, through the publication “Prevention and elimination of abuse, disrespect and maltreatment during childbirth in health institutions”, stated that these practices violate women’s rights to respectful care and constitute a threat to the right to life, health, physical integrity, equality, freedom, information and non-discrimination. This declaration mentions that physical and verbal abuse; humiliating practices; medical interventions performed without consent or in a coercive manner; lack of confidentiality and privacy; denial of pain relief; refusal of care in health services; and neglect against women in labor, constitute forms of disrespect and abuse in childbirth care [18].

Health professionals reject the term obstetric violence, not recognizing that violence can exist associated with labor and specifically at birth, legitimized by structural and symbolic power relations. The subjectivity of interpretation of obstetric violence refers to the complex dimension of this problem, since professionals, in the vast majority of situations, do not perceive them as violent, but as representative of their professional practice. On the other hand, many women, facing an event of greater vulnerability and without knowledge about their rights, do not identify certain manifestations of violence [19].

The World Health Organization itself, although recognizing the issue as an infringement of women’s rights to respectful care in labor, resists using the term obstetric violence, adopting the terms abuse, disrespect, and mistreatment in childbirth to describe the inappropriate relationship between the care team and women. These terms are often used synonymously; however, they have different definitions, which may make it difficult to clearly identify the problem [17]. However, despite the differences between the terminologies used, they all share common points by highlighting the abusive medicalization of the natural childbirth process, the roots in gender inequalities, the analogies with violence against women and the threat to their rights [20].

In the context of obstetric care, the authors conceptualize disrespect as the violation of women’s dignity, based on normative and discriminatory judgments, by health professionals and the resulting acts of omission or commission. Abuse refers to actions that increase the risk of harm to women’s health and well-being, reproduced through the practices of institutional medicine, which may or may not be intended to cause harm [21].

The terminology of disrespect and abuse, to endorse greater visibility on obstetric violence, as well as to allow problematizing it, as a barrier to safe and qualified childbirth care, was proposed by researchers who developed research in this area. They suggested a typology referring to different forms of disrespect and abuse in childbirth in health care facilities. The typology includes seven dimensions: (1) physical abuse; (2) non-consenting care; (3) nonconfidential care; (4) undignified care; (5) discrimination based on specific attributes of the woman; (6) neglect, denial, or negligence of care; and (7) detention in health facilities [22].

Other researchers, considering that the previous categories would be insufficient to characterize all forms of disrespect and abuse, have introduced the term childbirth mistreatment, which refers to women’s subjective experience in health systems, characterized by situations without care, impacting on their expectations regarding the labor and delivery process. The typification of maltreatment presented encompasses seven categories: (1) physical abuse; (2) sexual abuse; (3) verbal abuse; (4) stigma and discrimination; (5) failure to meet professional standards of care; (6) poor relationship between health professionals and women; and (7) health system conditions and limitations [23].

The concept of mistreatment is broader by allowing us to separate the issue of individual intentionality in violence and link it within the scope of quality in health [21]. Mistreatment, therefore, occurs as a form of structural violence, explained by the precarious conditions of health systems and the working conditions of their professionals, with the potential to reduce their ability to ensure the best possible care for women [23].

Since the presentation of the typology of childbirth abuse, the World Health Organization has adopted the term in its publications, considering it more acceptable and less provocative, which is still a questionable position, given its definition of violence that includes the acts of an intentional nature with the potential to cause harm, regardless of the result they produce [24]. Regarding how it is defined in the World Health Organization’s World Report on Violence and Health, the concept of violence refers to the intentional use of physical force or of actual or threatened power, against oneself, against another person, or against a group or community, resulting in or having any possibility of resulting in harm, injury, death, psychological harm, maldevelopment or deprivation [24].

Other authors also present an extension of the World Health Organization definition of violence, noting that intentionality as a central factor should not be applied to obstetric care, as it can have unintended and useless consequences to characterize obstetric care. The emphasis on intentionality is also contrasted in other studies that focus on the consequences of professionals’ choices, behaviors, and actions, rather than intentionality [25,26].

Warning about the adverse effect that the terminology of obstetric violence can have on health professionals, the World Health Organization also evokes the concept of respectful maternal care, framing it as a neglected component in the quality of obstetric care, but whose definition allows a less hostile approach to the problem. The term respectful maternal care focuses on the interpersonal relationships established between health professionals and women during childbirth care to preserve the dignity, confidentiality, and intimacy of the mother, to protect her from suffering, humiliation, and poor care practices, to allow her to make autonomous and informed choices, and to ensure continued support during labor [27].

The current debate around the issue of obstetric violence, as a form of gender violence, has increasingly aroused the interest and concern of national governments and international organizations, as well as social activist movements, in that it is recognized by the World Health Organization as a serious public health problem and a violation of human rights [28].

Recently, the World Health Organization presented recommendations for a positive birth experience based on safe, respectful, and quality care by health professionals. It highlights that respectful care centred on the woman, her accompaniment by a significant person, assertive communication, assistance by properly trained professionals, and the appropriate use of technology and effective interventions that promote the physiology of childbirth and ensure maternal-fetal well-being, are assumed as determinants in the positive and safe experience of childbirth [27].

Similarly, the United Nations General Assembly has reported the situation of violence against women in reproductive health services, associated with care at the time of birth, as a reality, proposing the adoption of strategies that enable women to live free of any form of violence, and ensure respect for their rights in the obstetric context [29].

The Sustainable Development Goals, through the third goal—“Ensure access to quality health care and promote well-being for all at all ages”—motivated the development of health policies worldwide, in order to increase the number of live births in health facilities and reduce maternal mortality, associated in many countries to neglect and neglect of care in the postpartum period. The fifth goal established—“Achieve gender equality and empower women and girls”—also aims to eliminate all forms of violence and discrimination against women, ensuring their reproductive rights, as well as their autonomy in decision-making [30].

In Portugal, following a survey, which revealed an excessive use of obstetric interventions during childbirth, as well as several cases that could be considered obstetric violence [31], the Portuguese parliament addressed a set of recommendations related to improving the quality of maternity care and the promotion of women’s rights in pregnancy and childbirth [32], and subsequently approved Law 110/2019, of 9 September, which grants rights to women in the context of sexual and reproductive health, as users, both in public and private health units. This law, while reaffirming the rights of women as progenitors, also increases medical liability in situations of obstetric violence, by providing specific rights for women, such as the right of the parturient to have minimal interference in her labor process [33].

The literature shows that although social movements for the humanization of childbirth have contributed to a greater relevance and visibility of the issue, and care practices based on scientific evidence have been officially systematized in the recommendations of national and international organizations to encourage the physiological dynamics of labor, respect for citizenship rights, and the nature of the human relationships between health professionals and women, different forms of obstetric violence continue to be reported in several studies developed worldwide. On the other hand, despite the fact that studies on the reality of obstetric violence during pregnancy, childbirth, and the puerperium period have increased, demonstrating the concern for its magnitude, there is no consensus on the appropriate terminology to name and define the situations of lack of adequate and satisfactory assistance based on respect for women’s dignity during childbirth [10,18,34].

The subjective nature of childbirth experience itself and the naturalization of violence rooted in the structures of health systems also lead researchers interested in the subject to face theoretical and methodological difficulties in defining terminology and measuring the object of study [35]. Different nomenclatures and definitions of this construct cause lack of precision in the prevalence of such acts of violence, difficulty in comparing the realities of different countries, and there is a scarcity of studies focusing on the possible negative outcomes of this problem for the health of women and their newborns [17].

The lack of international unanimity on how the complexity of this phenomenon can be scientifically defined, determines that the World Health Organization advocates the need for further research that can lead to a better conceptualization and understanding of disrespect and abuse of women during childbirth, as well as ways to prevent and eliminate these types of violence [18].

As nurse midwives and researchers, in an attempt to better understand what is contemplated in the practice of care, as a configuration of obstetric violence, we feel the need for analysis, refinement and clarification of the concept by identifying its characteristics and dimensions, namely: terminology, typology, nature and predisposing factors, as well as its consequences for women. This review aims to analyze the phenomenon of obstetric violence, which affects women in labor, using the scoping review [36] methodology, as it is considered the best choice in situations where there is interest in identifying concepts, mapping them, analyzing, reporting or discussing them [37].

This concept analysis will be subsequently carried out under the evolutionary view of the Rodgers’ method, in order to understand how obstetric violence associated with labor is characterized. In the literature, Rodgers’ evolutionary method of conceptual analysis is characterized as an inductive and descriptive model, applied to investigate the history of a given concept. This method is structured in six steps: (1) defining the concept of interest and related expressions; (2) selecting the field for data collection; (3) analyzing the essential attributes or characteristics of the concept; (4) analyzing the contextual basis of the concept (antecedents and consequents); (5) identifying, if necessary, an example of the concept to be investigated, and (6) determining the implications and hypotheses for the concept [38]. According to this model, the concept is understood in the particularity of its context, from a dynamic perspective, and it changes over time, i.e., concepts are dynamic and context-dependent. In other words, it is a cyclical process, in which the meaning of a concept depends on its use and application [39].

### Objective

The main objective of this scoping review is to analyze the concept of obstetric violence related to the care of women during labor.

## 2. Materials and Methods

This is a protocol study for a scoping review, which will be the starting point for carrying out the analysis of the concept of obstetric violence, according to Rodgers’ conceptual method. A scoping review or scoping study follows a systematic approach to map the main concepts about a given area of knowledge; examine their extent in research; identify knowledge gaps in existing research; and consequently, contribute an overview of the available evidence in the literature [40,41]. In this case, a scoping review will be conducted on published research whose object of study focuses on the concept of obstetric violence, more specifically on its characteristics and dimensions. The methodology will be based on the guidelines proposed by the Joanna Briggs Institute [36,42], adopting five research steps: (1) identification of the research question; (2) identification of relevant studies; (3) selection of studies; (4) data extraction; and (5) compilation, summary, and reporting of results.

This protocol will be guided by the guidelines of the Preferred Reporting Items for Systematic Reviews and Meta-Analyses extension for Scoping Reviews (PRISMA-ScR) [42].

Drafting of the protocol began in March 2022 and the scoping review is expected to be completed in September of the same year. This protocol is registered in the Open Science Framework platform with the following registration number: 10.17605/OSF.IO/U46B5.

### 2.1. Step 1—Identification of the Research Question

In the scoping review protocol, we defined the following research question, adapting the acronym PCC (Population, Concept, Context) [18]: How does one characterize the concept of obstetric violence (Concept), in women’s care (Population), in the context of labor (Context)?

### 2.2. Step 2—Identification of Relevant Studies

The research strategy will focus on the search for published studies. We proceeded to the identification of search descriptors according to the Health Science Descriptors [43] that would answer the research question. In this research, the following descriptors will be used: “obstetric violence”; “woman” and “parturition”. The natural and indexed terms for these descriptors, in the chosen databases, will also be applied to allow the identification of relevant studies, combining them by means of the boolean operators AND and OR, according to the specifications of each database. The search strategy is presented in Appendix A.

According to the PCC question (Population, Concept and Context), the following inclusion and exclusion criteria for the research studies were established (Table 1).

The electronic search will be performed using the search engine EBSCOhost Research Platform (selecting CINAHL Complete and MEDLINE Complete databases); in health databases—Virtual Health Library (VHL), PubMed and SciVerse Scopus—and in the Open Access Scientific Repository of Portugal.

### 2.3. Step 3—Study Selection

The selection of studies to be included in the scoping review will meet the eligibility criteria previously mentioned. The studies identified in the search will be entered into EndNote software, so that duplicates are removed. In a first stage, the selection of studies will be performed by two independent researchers, by reading the title, abstract and keywords, and in case of doubt, the full text, excluding those that do not meet the defined inclusion criteria. If there is any disagreement or doubt between the two reviewers, a tie-breaker will be established by a third reviewer. In the next step, the full text will be read independently by both reviewers.

The inclusion of any study will be conditioned by the prior application of the critical appraisal instruments made available by the Joanna Briggs Institute.

The results obtained in the research, as well as the selection process of the studies, will be subsequently reported in the scoping review and presented in the form of a Prisma Scoping Review^®^ [42] flowchart.

### 2.4. Step 4—Data Extraction

The extraction of data from the selected studies will be carried out using a proprietary tool (Appendix B), designed to identify specific data about the population, concept, context, methodology, and results that are relevant to the objective under study. If necessary, during the data extraction process, this tool will be subject to review and modification.

Any disagreements that arise will be resolved through discussion or with the support of a third party researcher. Where appropriate, article authors will be contacted to request missing or additional data.

### 2.5. Step 5—Compiling, Summarizing and Reporting the Results

The extracted data will be included in a table format for summarized presentation of the results. The table will contain the title of the study; author(s)/year of publication; type of study; objective(s); population; methodology; results and main conclusions and findings, according to the research question (Appendix C). In the discussion of the main results, a narrative approach will be used to organize and categorize the main findings on the mapping of the characteristics and dimensions of the concept of obstetric violence. The Vancouver standard will be used, as recommended by the Joanna Briggs Institute.

Since this scoping review searches for evidence of published studies to obtain secondary data, no ethical approval will be required for the implementation of this study.

## 3. Discussion

Labor and delivery are natural processes of human reproduction; however, they are unique for each woman due to the many challenges this experience brings. The environment in which a woman experiences childbirth can have a decisive influence on this personal experience and, consequently, on her overall well-being [44].

The existence of situations involving dehumanized care, communication failures, disempowerment, abuse of medicalization, and pathogenesis of reproductive physiological processes are just some examples of situations that may fall under the concept of obstetric violence, which impact the experience of labor and birth [45]. Recently, a multi-country study developed by the World Health Organization, aiming to assess the quality of maternal and neonatal care, revealed that women, during the labor process, are subjected to various forms of abuse, disrespect, and violence [46].

The problem is recognized by several entities and organizations in the health field and, despite being the focus of numerous studies that reveal its magnitude, it still represents a critical and controversial issue, remaining confusion and lack of consensus around the terminology and experiences associated with this phenomenon.

The term obstetric violence has, therefore, very strong significant connotations, with the ability to surprise many people; truly embarrass health professionals qualified for childbirth care; and provoke widespread rejection by professionals from different areas [15].

## 4. Strengths and Limitations of the Study

It is our intention that the scoping review, based on the protocol presented, by allowing an analysis of the characteristics and dimensions of the concept of obstetric violence, in the context of assistance to women in labor, will produce results that will support academic research on the subject. On the other hand, we hope to enhance the expansion of the debate around the problem, especially with regard to the reality of our country.

This review protocol may also be an instrument of guidance for future research on the conceptualization of obstetric violence.

Due to the significant amount of existing information on the approach to the concept, it was considered that the most appropriate methodology was the search for studies in the last 10 years, which may still be a limitation if there is information considered relevant whose publication is not included in this time limitation.

## Figures and Tables

**Table 1:** Inclusion criteria table.

Strategy	Inclusion Criteria
Population	Studies whose population includes women who have experienced labor in a hospital setting, either public or private.
Concept	Studies that address the dimensions of the concept of obstetric violence (terminologies; typologies; causes or nature; predisposing or determining factors; and consequences).
Context	Studies regarding the assistance to the labor process, in public and/or private hospitals.
Other criteria	Studies in Portuguese, English or Spanish. Studies of a qualitative and quantitative nature, of all types, whether experimental, quasi-experimental, literature reviews, meta-analyses, theses and dissertations. Studies published in the last 10 years (2012–2022). Articles with more than 75% in the quality criteria of the Joanna Briggs Institute tools.
	**Exclusion Criteria**
	Articles with less than 75% of the quality criteria, considering the tools of Joanna Briggs Institute.

## Data Availability

Not applicable.

## Appendix A. Search Strategy

**Database**	**Search Strategy**
CINAHL Complete MEDLINE Complete	[(obstetric violence OR abuse in childbirth OR disrespect and abuse in childbirth) AND (woman OR women) AND (parturition OR delivery OR childbirth OR birth)]
Vitual Health Library (VHL)e	
PubMed	
SCOPUS	
Open Access Scientific Repository of Portugal	

## Appendix B. Data Extraction Tool

Data Extraction Tool	
Review Title Analysis of the concept of obstetric violence: Scoping Review	
Overall review objective To analyse the concept of obstetric violence related to the care of women during labor.	
Review Question How does one characterize the concept of obstetric violence, in the care of women, in the context of labor?	
Inclusion Criteria Studies whose population is composed of women who have experienced labor; studies that address the concept of obstetric violence, in the context of care assistance in the labor process; studies referring to the hospital context (public and private); studies of qualitative and quantitative nature, in Portuguese, English and Spanish languages. Articles with more than 75% in the quality criteria in the JBI instruments. Exclusion Criteria Articles that are not made available with full text. Articles with less than 75% of the quality criteria, considering the JBI tools.	
Details and characteristics of the study	
Title, Authors, Year and Country	
Population	
Context	
Concept (related and replacement terminology)	
Attributes of the concept (nature, causes and predisposing factors)	
Contextual basis of the concept (background and consequences)	
Coments	

## Appendix C. Table for the Presentation of Results

**Title, Authors, Year and Country**	**Goal**	**Type of Study**	**Population**	**Methodology**	**Results**	**Conclusions and Main Findings**
